# Elastic constants of fibre-textured thin films determined by X-ray diffraction

**DOI:** 10.1107/S0021889809011807

**Published:** 2009-05-02

**Authors:** K. J. Martinschitz, R. Daniel, C. Mitterer, J. Keckes

**Affiliations:** a Department of Materials Physics, University of Leoben and Erich Schmid Institute for Materials Science, Austrian Academy of Science, Jahnstrasse 12, 8700 Leoben, Austria; b Department of Physical Metallurgy and Materials Testing and Christian-Doppler Laboratory for Advanced Hard Coatings, University of Leoben, Austria

**Keywords:** X-ray diffraction, thin films, elastic constants, fibre texture

## Abstract

A new methodology is presented that allows the rapid determination of elastic constants of cubic fibre-textured thin films by X-ray diffraction. The theoretical concept is developed and tested on calculated examples of Cu and CrN films. The mechanical elastic constants are extrapolated from X-ray elastic constants by taking into consideration crystal and macroscopic elastic anisotropy. The derived algorithm enables the determination of a reflection and the corresponding value of the X-ray anisotropic factor Γ for which the X-ray elastic constants are equal to their mechanical counterparts in the case of fibre-textured cubic polycrystalline aggregates. The approach is independent of the crystal elastic anisotropy and depends on the fibre-texture type, the texture sharpness, the number of randomly oriented crystallites and the supposed grain-interaction model. In the experimental part, out-of-plane Young’s moduli of 111 and 311 fibre-textured Cu and CrN thin films deposited on monocrystalline Si(100) substrates are determined. The moduli are extrapolated from thin-film experimental X-ray elastic constants that are determined by a combination of X-ray diffraction substrate curvature and sin^2^ψ methods. For the calculation, the film macroscopic elastic anisotropy (texture) is considered. The advantage of the new technique lies in the fact that experimental moduli are determined nondestructively, using a static diffraction experiment, and represent volume-averaged quantities.

## Abstract 2

Supposing the Hill grain-interaction model, it is demonstrated that X-ray elastic constants can be used to determine mechanical elastic constants of cubic fibre-textured thin films. The new approach is demonstrated by the experimental characterization of out-of-plane moduli of fibre-textured Cu and CrN thin films.

## 1. Introduction

X-ray elastic constants and diffraction stress factors are usually used to calculate residual stresses from experimental X-ray elastic strains (Dölle, 1979 ▶; Noyan & Cohen, 1987 ▶; Welzel *et al.*, 2005 ▶). In the case of a specimen with crystal elastic anisotropy, the X-ray elastic constants differ for various *hkl* reflections and are, moreover, dependent on the texture, the grain-interaction mechanism and the single-crystal elastic constants (Barral *et al.*, 1987 ▶; van Houtte & De Buyser, 1993 ▶; van Leeuwen *et al.*, 1999 ▶; Leoni *et al.*, 2001 ▶; Badawi *et al.*, 2002 ▶; Welzel, 2002 ▶; Welzel *et al.*, 2005 ▶).

Numerous experimental as well as computational methods have been developed in the past few decades to determine mechanical elastic moduli (or even single-crystal elastic constants) from diffraction elastic constants (Hayakawa *et al.*, 1985 ▶; Humbert & Diz, 1991 ▶; Wright, 1994 ▶; Gnäupel-Herold *et al.*, 1998 ▶; Howard & Kisi, 1999 ▶; Badawi *et al.*, 2002 ▶; Badawi & Villain, 2003 ▶). In the case of experimental characterization using X-rays or neutrons, polycrystalline samples (bulk or thin films, usually on a flexible substrate) are *in-situ* strained, and diffraction elastic strains are recorded and correlated with the applied stress. To extract the mechanical elastic constants, Reuss, Voigt, Hill and Eshelby–Kröner grain-interaction models are usually applied (Hayakawa *et al.*, 1985 ▶; Wright, 1994 ▶; Howard & Kisi, 1999 ▶; Faurie *et al.*, 2006 ▶). Although the grain-interaction models represent only idealized theories, the elastic constants of quasi-isotropic or textured samples, or samples with crystal elastic anisotropy, have been obtained with a relatively good accuracy (Howard & Kisi, 1999 ▶; Villain *et al.*, 2002 ▶; Badawi & Villain, 2003 ▶; Goudeau *et al.*, 2004 ▶). It was found that the anisotropic Hill model is a good approximation of the experimental data obtained from polycrystalline samples (usually within the precision of the techniques applied) and, moreover, the more sophisticated models were usually very close to the Hill approximation (Howard & Kisi, 1999 ▶, p. 632). In the majority of cases (Humbert & Diz, 1991 ▶; Wright, 1994 ▶; Gnäupel-Herold *et al.*, 1998 ▶; Howard & Kisi, 1999 ▶; Badawi *et al.*, 2002 ▶; Badawi & Villain, 2003 ▶), however, the experimental elastic moduli or single-crystal elastic constants are obtained from *in-situ* experiments coupling diffraction and sample loading, *i.e.* destructively, whereby it is necessary to use a tensile stage.

Recently, a new rapid experimental approach based on the simultaneous application of sin^2^ψ and X-ray diffraction substrate curvature techniques was proposed (Eiper *et al.*, 2005 ▶, 2006 ▶; Keckes *et al.*, 2007 ▶; Martinschitz *et al.*, 2006 ▶). The new approach provides an opportunity to quantify experimental X-ray elastic strains and macroscopic stresses in thin films using a static diffraction experiment. The stresses applied on the film are determined from the geometrical changes of the elastically deformed substrate that is attached to the film (Stoney, 1909 ▶; Segmüller *et al.*, 1989 ▶). The experimental stress and strain can then be used to evaluate experimental X-ray elastic constants and stress factors (Eiper *et al.*, 2005 ▶, 2006 ▶; Martinschitz *et al.*, 2006 ▶).

Mechanical elastic constants can be extrapolated from X-ray elastic constants considering crystal and macroscopic elastic anisotropy. In the case of cubic polycrystalline aggregates with macroscopic elastic isotropy (quasi-isotropic materials) that obey the Hill grain-interaction model, it was demonstrated that X-ray elastic constants correspond to their mechanical counterparts for 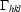 = 0.2, where 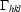 is the X-ray anisotropic factor according to the Reuss grain-interaction model, given by
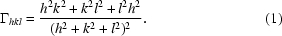
(*hkl*) are Miller indices of a crystallographic plane (Bollenrath *et al.*, 1967 ▶). According to the Reuss (1929 ▶) model, X-ray elastic anisotropy is often expressed as a function of 3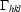, and this formalism will be applied hereafter.

It is the aim of this paper to analyse under which conditions knowledge of X-ray elastic constants can be used to determine or estimate mechanical elastic constants of cubic fibre-textured thin films that obey the Hill grain-interaction model. Firstly, the mechanical elastic constants of Cu and CrN will be calculated using a Hill model that represents a reasonable simplification of the problem (Hill, 1952 ▶; Bunge & Roberts, 1969 ▶; Bunge, 1982 ▶; Gnäupel-Herold *et al.*, 1998 ▶; Howard & Kisi, 1999 ▶; Leoni *et al.*, 2001 ▶; Welzel, 2002 ▶). As a next step, the mechanical values will be compared with calculated X-ray elastic constants. As a result, a 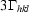-dependent selection rule will be derived, where the subscript *hkl* in 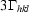 denotes a reflection for which mechanical and X-ray elastic constants are equal. The approach will be demonstrated by experimental characterization of out-of-plane moduli of fibre-textured Cu and CrN thin films. The moduli are extracted from experimental X-ray elastic constants that are determined by a combination of X-ray diffraction substrate curvature and sin^2^ *ψ* methods in a static diffraction experiment. It should be noted that the methodology derived in this paper can be generally applied to any equibiaxially loaded or stressed polycrystalline aggregate with the fibre texture oriented perpendicular to the stress direction.

## 2. Mechanical elastic constants of thin films

### 2.1. Hill grain-interaction model

**Figure 1:**
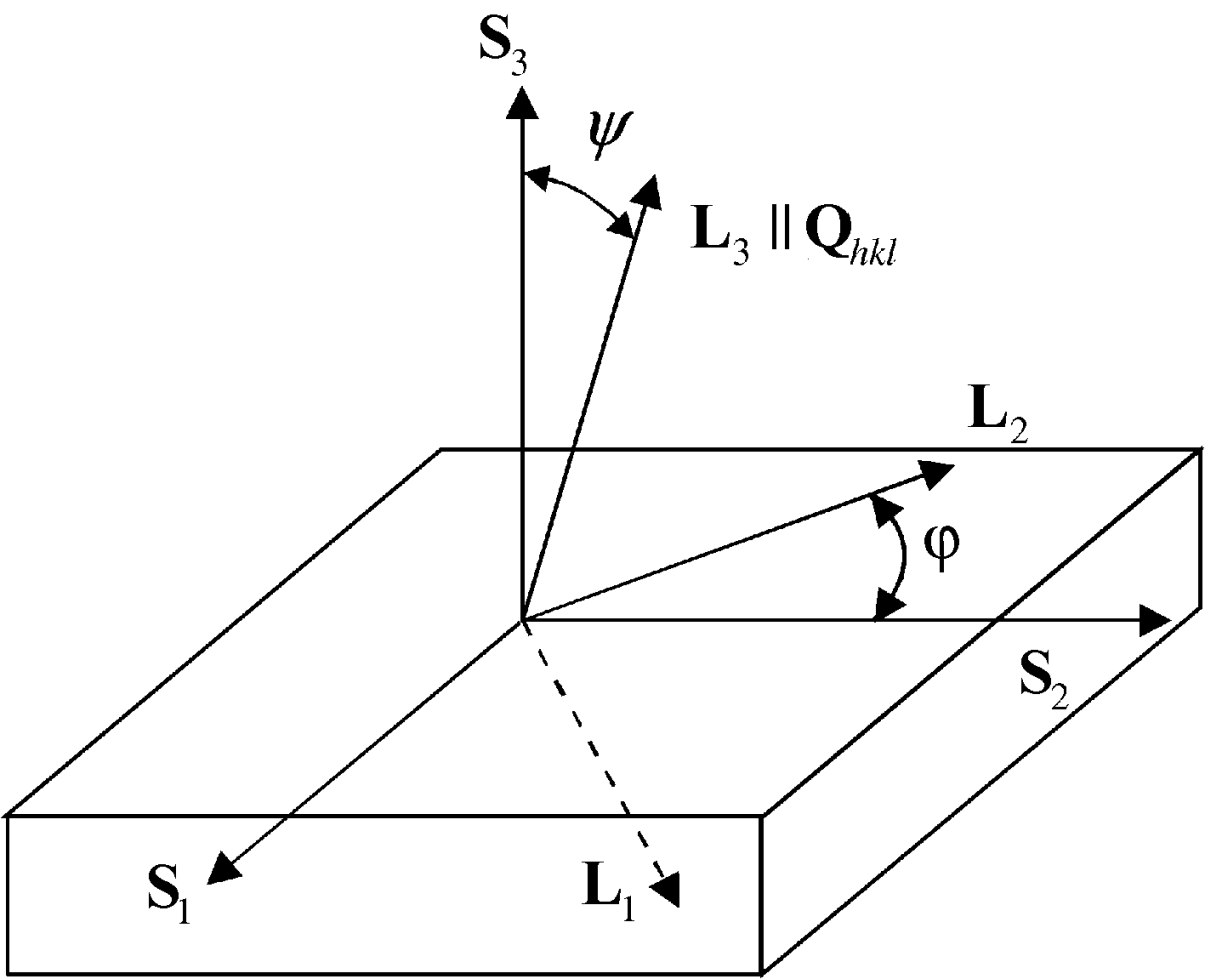
The definition of the two coordinate systems used for the characterization of in-plane elastic strains using the sin^2^ψ method: sample system S and laboratory system L (Noyan & Cohen, 1987 ▶). The X-ray elastic strain along the direction 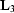 (which is parallel to the diffraction vector 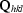) is characterized by measuring the reflection *hkl*. The orientation of the vector 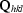 with respect to 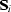 is defined by the angles ϕ and ψ. The direction cosines *a*_*ij*_ in equation (6) represent a transformation from S to L coordinate systems.

Elastic behaviour of a thin film deposited on a solid substrate can be represented by Hooke’s law: 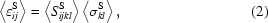
where 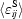 is the mechanical elastic strain, 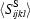 expresses the mechanical elastic constants of the film and 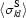 represents the residual stress (Nye, 1976 ▶; Suresh & Freund, 2003 ▶). The brackets 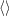 denote volume averages for all crystallites (*i.e.* mechanical averages; Welzel, 2002 ▶). The stress, strain and compliance tensors in equation (2) are expressed in the sample coordinate system (S) (Fig. 1 ▶).

In general, 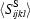 of a polycrystalline film depends on the texture, the single-crystal elastic constants and the grain-interaction mechanism (van Houtte & De Buyser, 1993 ▶). In practice, the Hill grain-interaction model can be used to evaluate 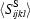 of the film (Hill, 1952 ▶) using the arithmetic mean of the compliance tensors 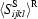 and 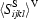 obtained from the Reuss and Voigt grain-interaction models: 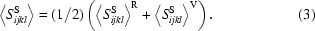
Elastic constants according to the Reuss average 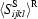 can be calculated as follows:
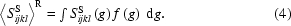
In the case of the Voigt average, 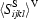 can be determined according to
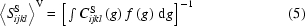
where *f*(*g*) represents the orientation distribution function (ODF) of the crystallites in the film (Bunge, 1982 ▶; Huang & Weaver, 2005 ▶). 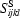 and 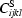 in equations (4) and (5) are single-crystal elastic constants expressed in S, while *f*(*g*)d*g* indicates the volume fraction of the crystallites with the orientation *g*. The integration in equations (4) and (5) is carried out over the whole ODF space (van Houtte & De Buyser, 1993 ▶).

The tensor 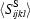 [equation (2)] represents the elastic behaviour of the material in the sample coordinate system S (Fig. 1 ▶) (Nye, 1976 ▶) and can be expressed in the L system using 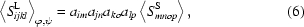
where 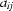 represent the direction cosines between the L and S systems (Fig. 1 ▶; Noyan & Cohen, 1987 ▶).

In practice, Young’s modulus 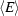 is usually used to express elastic behaviour of materials. The magnitude of 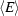 in the direction 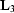 can be obtained from the tensor 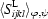: 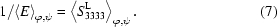
The out-of-plane Young’s modulus 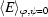 can be obtained from equation (7) using 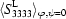.

### 2.2. Calculation of mechanical elastic constants

Using the procedure from the previous section, Young’s moduli of Cu and CrN thin films with various fibre textures were calculated numerically, applying single-crystal elastic constants from Table 1 ▶ and various types of ODFs.

**Figure 2:**
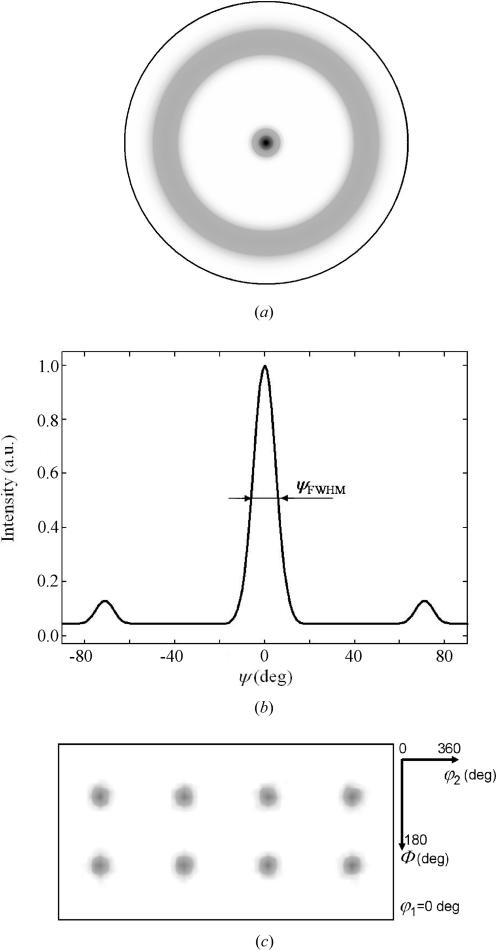
(*a*) A simulated 111 pole figure of a cubic thin film with a strong 111 fibre texture and a 10% fraction of randomly oriented crystallites. (*b*) The distribution of the intensity across the pole figure, where the variable ψ_FWHM_ = 10° represents the sharpness of the texture. (*c*) A representative ϕ_1_ = 0° section of the ODF, which is identical for all ϕ_1_ values, documents the 111 fibre character of the texture (Bunge, 1982 ▶).

In Fig. 2 ▶, an example of a 111 pole figure, the distribution of the intensity across the pole figure and the corresponding ODF demonstrate a strong 111 fibre texture with a 10% fraction of randomly oriented crystallites in a cubic thin film.

As a parameter for the ODF calculation, the full width at half-maximum at the centre of the pole figure [which is usually measured experimentally using a ψ scan (Bunge, 1982 ▶)], hereafter denoted ψ_FWHM_, was used (Fig. 2 ▶). Since the aim is to develop a simple laboratory method to determine elastic constants of thin films, ψ_FWHM_ was used as a measure of the texture sharpness [instead of using variables expressed in terms of the ϕ_1_, Φ and ϕ_2_ angles (Fig. 2 ▶), which are usually needed to define ODF properties according the Bunge (1982 ▶) notation]. Numerous ODFs with ψ_FWHM_ in the range 0–180° with a step of 5° were generated in order to calculate 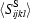 [equation (2)] and subsequently the out-of-plane Young’s modulus 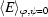 [equation (7)]. This calculation was performed for various *uvw* fibre textures, where the subscript *uvw* represents the indices of the (*uvw*) crystallographic planes oriented preferably parallel to the sample surface. Additionally, it was supposed that the films also contain crystallites with a random orientation (hereafter denoted as ISO) in the range 0–100%.

**Figure 3:**
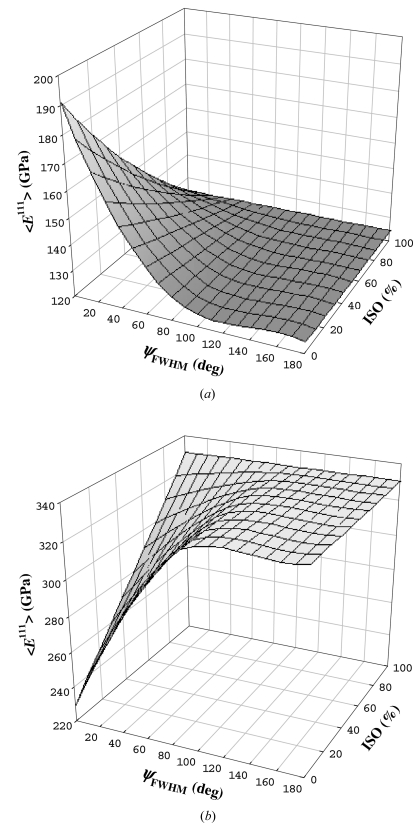
Calculated out-of-plane Young’s moduli 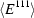 of 111 fibre-textured Cu (*a*) and CrN (*b*) thin films as a function the texture sharpness ψ_FWHM_ (Fig. 2 ▶) and ISO.

As an example of the procedure, calculated out-of-plane Young’s moduli 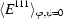 of Cu and CrN thin films with 111 fibre texture are presented in Fig. 3 ▶. As parameters for the calculation, the texture sharpness ψ_FWHM_ and the number of randomly oriented crystallites ISO were applied. The three-dimensional plots document that the moduli of the film exhibit relatively strong maxima or minima for small ψ_FWHM_ and ISO but converge to the moduli of isotropic Cu and CrN when one of the parameters increases.

Cu and CrN possess different types of crystal elastic anisotropy (Table 1 ▶), with the Zener (1948 ▶) anisotropy ratio ZAR defined as 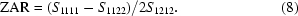
Since the 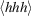 direction in Cu is stiffer than all others, the films with less pronounced 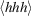 textures exhibit smaller moduli (Fig. 3 ▶). In CrN, the opposite situation has to be considered.

**Figure 4:**
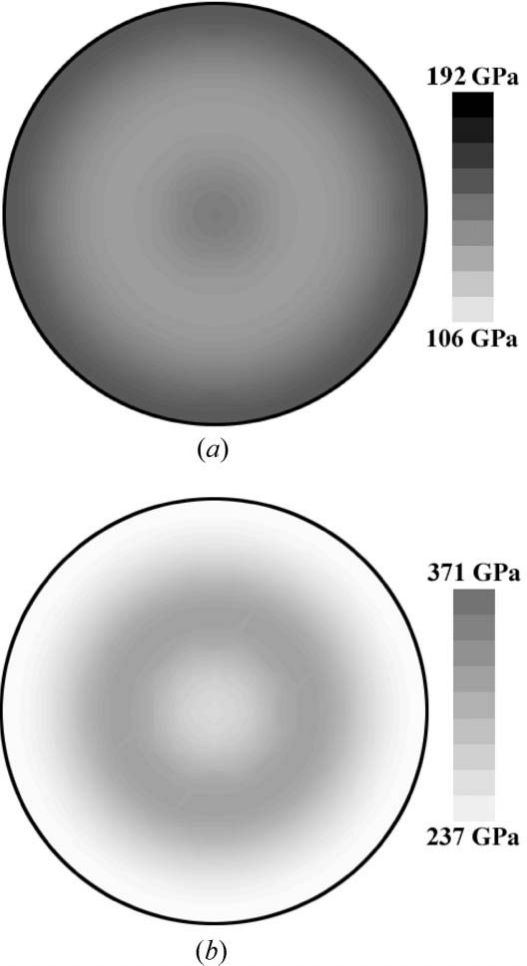
Calculated Young’s moduli 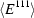 of 111 fibre-textured Cu (*a*) and CrN (*b*) expressed in polar coordinates ϕ and ψ (Fig. 1 ▶). The moduli were calculated using the ODF from Fig. 2 ▶. The outer ring corresponds to 90°.

The results in Fig. 3 ▶ represent out-of-plane Young’s moduli calculated from 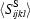. In the case of fibre-textured thin films, however, the elastic behaviour is in-plane isotropic (*i.e.* independent of the angle ϕ) but dependent on the tilt angle ψ (Fig. 1 ▶). In order to demonstrate this situation, Young’s moduli of CrN and Cu 111 fibre-textured thin films (Fig. 2 ▶) were calculated as a function of angles ϕ and ψ using equations (3)–(7) [Disp-formula fd5] [Disp-formula fd6] [Disp-formula fd7] and are presented in polar coordinates in Fig. 4 ▶. The difference in crystal elastic anisotropy causes the CrN modulus to possess a minimum at ψ = 0°, in contrast to the Cu dependence, which exhibits a maximum at the centre of the polar plot (Fig. 4 ▶).

## 3. X-ray elastic constants of thin films

### 3.1. X-ray elastic moduli

In X-ray diffraction, Hooke’s law relates X-ray elastic strain 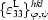 measured in the direction 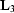 by scanning the reflection *hkl*, X-ray elastic compliances 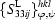 and the macroscopic stress 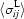 expressed in the L coordinate system (Fig. 1 ▶) as follows: 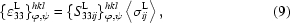
where 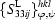 depends generally on the texture, the grain-interaction mechanism, the reflection *hkl*, the single-crystal elastic constants, and the angles ϕ and ψ (Dölle, 1979 ▶; van Houtte & De Buyser, 1993 ▶). The brackets 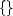 denote volume-weighted averages for all diffracting crystallites (*i.e.* diffraction averages; Welzel, 2002 ▶). For simplicity, 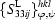 can be calculated using the Hill grain-interaction model as follows (Serruys, 1988 ▶; van Houtte & De Buyser, 1993 ▶):
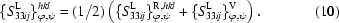
The X-ray elastic compliances 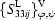 represent an elastic behaviour of the film according the Voigt grain-interaction model (V) and can be calculated as 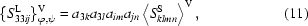
where the 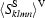 tensor was obtained using equation (5) (van Houtte & De Buyser, 1993 ▶).

X-ray elastic compliances according the Reuss grain-interaction model (R) can be obtained by integration over the crystal orientations *g* for which the diffraction vector 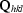 is parallel to the direction 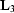 (van Houtte & De Buyser, 1993 ▶): 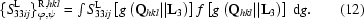
Considering fibre-textured cubic thin films with the fibre axis oriented perpendicular to the sample surface, it will be supposed that

(i) The mechanical state of the films is biaxial and in-plane isotropic with 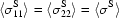 and 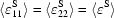. Moreover, shear stresses 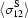 and 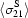, shear strains 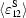, 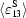 and 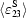, and out-of-plane stresses 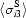 can be neglected on the macroscopic scale.

(ii) The thin films are in-plane elastic isotropic, and not only the distribution of crystallites but also the grain-interaction mechanism possess a rotational symmetry. The elastic properties of the films are therefore not dependent on the azimuth angle ϕ, with 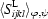 = 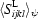.

The above implies that equation (9) can be expressed as follows (Stickforth, 1966 ▶): 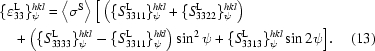
In the case of the experimental dependence of 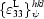 on 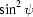, the term 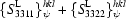 corresponds to the intercept on the 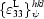 axis and the term 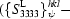 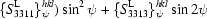 is responsible for the curvature in the 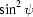 plots. The term 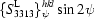 vanishes, however, under certain conditions (*cf*. Stickforth, 1966 ▶; van Houtte & De Buyser, 1993 ▶; Welzel, 2002 ▶).

Since the tensor components 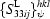 in equation (13) change as a function of the orientation of the diffraction vector 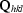, they can be used to determine diffraction elastic constants as a function of (*hkl*) and ψ. For example, the diffraction modulus 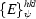 along the direction 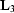 reads 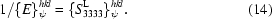
On condition that the 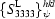 components are independent of the angles ψ and ϕ and the material is quasi-isotropic, equation (13) can be written as 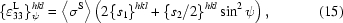
in which the symbols 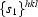 and 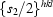 represent isotropic X-ray elastic constants (Dölle, 1979 ▶; Noyan & Cohen, 1987 ▶). The constants are sometimes substituted as (Noyan & Cohen, 1987 ▶) 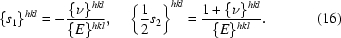
The symbol 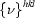 represents the diffraction Poisson number determined by the measurement of reflection *hkl*. In the case of macroscopic elastic isotropic aggregates, 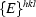 and 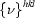 can be calculated using equations (15) and (16) provided 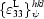 and 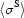 are known.

### 3.2. Calculation of diffraction elastic moduli

**Figure 5:**
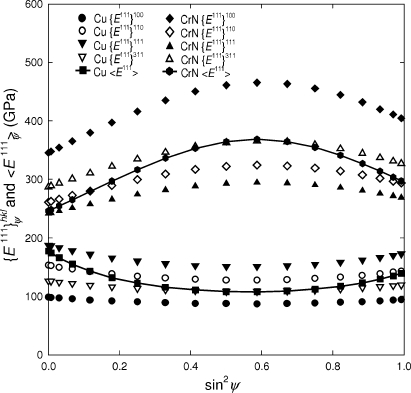
Diffraction 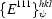 and mechanical 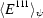 Young’s moduli of Cu and CrN films with 111 fibre texture, shown as a function of the sample tilt angle ψ. The moduli were calculated using the ODF from Fig. 2 ▶ supposing the Hill (1952 ▶) grain-interaction model. The diffraction moduli 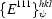 represent the elastic response of diffracting grains and the mechanical moduli 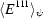 represent the elastic response of all crystallites in the film. The moduli are expressed as a function of the tilt angle ψ, which defines also the orientation of the diffraction vector 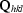 (Fig. 1 ▶).

The X-ray elastic compliances 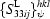 express the elastic behaviour of the aggregate along the diffraction vector 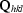. In Fig. 5 ▶, Young’s moduli of 111 fibre-textured Cu and CrN thin films are presented as a function of the tilt angle ψ.

The data in Fig. 5 ▶ document that the mechanical moduli 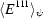 of the 111 fibre-textured films lie always between diffraction moduli 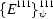 and 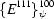. The diffraction moduli 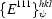 represent the elastic response of *diffracting crystallites* in the direction of diffraction vector 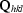 (Fig. 2 ▶). The mechanical moduli 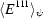 represent the elastic response of *all crystallites* in the direction of diffraction vector 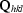. For ψ = 0 the out-of-plane mechanical modulus 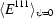 approaches the diffraction modulus 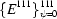 (which can be obtained by the characterization of the 111 reflection and 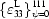) because of the specific texture type (Fig. 5 ▶).

## 4. A comparison of mechanical and X-ray elastic constants

### 4.1. General considerations

The results in Fig. 5 ▶ demonstrate that the mechanical elastic constants 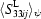 are constrained by the X-ray elastic constants 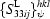. It is therefore obvious that, by considering a specific ODF, tilt angle ψ and single-crystal elastic constants, it is always possible to determine a reflection *hkl* and a corresponding X-ray anisotropy factor 3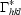 for which the X-ray elastic constants are equal to their mechanical counterparts (Fig. 5 ▶). 3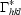 will therefore be used to denote conditions in accordance with the Hill model, under which 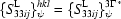 = 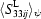.

### 4.2. Isotropic case

In the case of polycrystalline materials with crystal elastic isotropy or with negligible macroscopic elastic anisotropy, 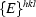 and 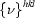 as well as 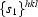 and 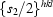 are independent of the angle ψ, and equation (15) supposes a linear dependence of 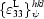 on sin^2^ψ (Stickforth, 1966 ▶; Noyan & Cohen, 1987 ▶; van Houtte & De Buyser, 1993 ▶). Provided that the elastic strain 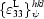 and the macroscopic stress 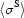 can be determined by experiment, the isotropic X-ray elastic constants 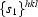 and 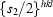, and subsequently also 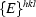 and 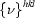, can be obtained by solving a system of linear equations of the same type as equation (15) when 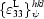 is known for different ψ (Ortner, 1986*a* ▶,*b* ▶).

**Figure 6:**
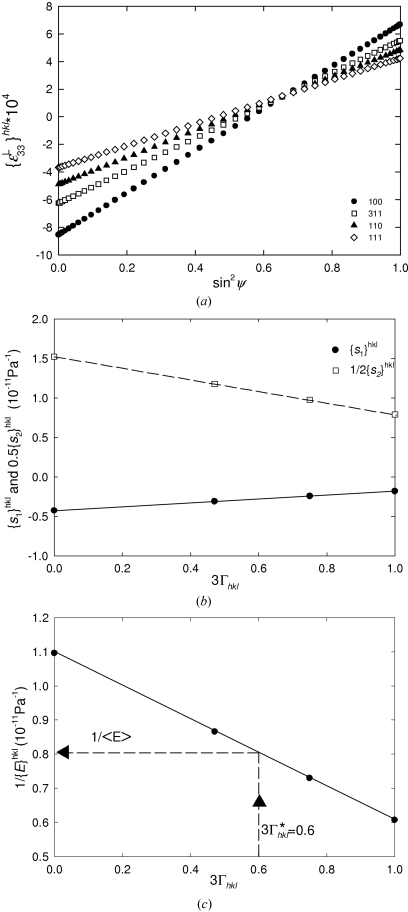
(*a*) Calculated X-ray elastic strains in a quasi-isotropic Cu thin film with equibiaxial stress of 100 MPa. (*b*) X-ray elastic constants 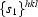 and 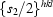 refined from (*a*), plotted as a function of 3*Γ*_*hkl*_. (*c*) Reciprocal diffraction Young’s moduli 1/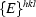 obtained from (*b*). The mechanical modulus 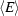 can be extrapolated for 3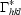 = 0.6, resulting in a value of 123.45 GPa.

An example of this procedure is presented in Fig. 6 ▶. Considering the single-crystal elastic constants from Table 1 ▶ and an in-plane isotropic stress 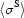 = 100 MPa, calculated diffraction strains 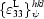 for a quasi-isotropic Cu thin film are presented in Fig. 6 ▶(*a*).

According to equation (15), the slopes in Fig. 6 ▶(*a*) correspond to 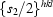 and the intercepts on the 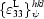 axis can be correlated with the magnitude of 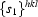. In practice, the X-ray elastic constants are obtained by fitting the experimental data from Fig. 6 ▶(*a*) using equation (15). The reciprocal diffraction elastic moduli 1/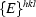 in Fig. 6 ▶(*c*) can then be determined from 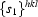 and 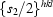 (Fig. 6 ▶ *b*) as follows: 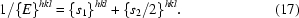
The reciprocal mechanical modulus 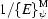 = 0.81 × 10^−11^ Pa^−1^ was extrapolated from the reciprocal diffraction moduli 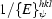 supposing 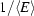 = 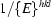 for 3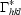 = 0.6, as predicted by the Hill grain-interaction model for quasi-isotropic materials (Bollenrath *et al.*, 1967 ▶). The mechanical modulus 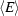 is therefore 123.45 GPa. This procedure is, however, valid only in the case of elastic isotropic aggregates.

### 4.3. Fibre-textured thin films

The procedure described in Fig. 6 ▶ is an often used simplification. Polycrystalline thin films are, however, usually macroscopic elastic anisotropic, and therefore the extrapolation of the mechanical modulus from X-ray elastic constants for 3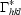 = 0.6 would provide incorrect results.

In the majority of cases, polycrystalline thin films possess a certain *uvw* fibre texture with the fibre axis oriented perpendicular to the substrate surface. In that case, the mechanical and X-ray elastic compliances are dependent on the angle ψ (Figs. 4 ▶ and 5 ▶). In order to determine the experimental 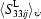 from 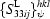 it is necessary to know the exact value of 3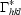, which is also dependent on ψ, as demonstrated in Fig. 5 ▶. We discuss below the possibilities for determining 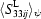 and 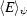 from the experimental 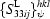 by applying Hooke’s law [equation (13)].

(i) In the case of in-plane elastic isotropic films 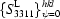 is equal to 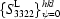 for ψ = 0 and equation (13) reduces to 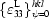 = 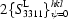. 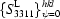 can be determined experimentally by evaluating the intercept of the sin^2^ψ dependence on the 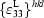 axis when 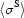 is known. The dependence of 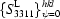 on 3Γ_*hkl*_ could then be used to determine the thin-film mechanical compliance 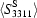.

(ii) By comparing the intercepts 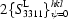 and the slopes 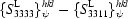 of the sin^2^ψ curves for 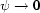 [and by simultaneously neglecting the term 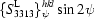 since 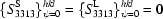 for hexagonal macroscopic symmetry of the sample (Martinschitz, 2008 ▶)], equation (13) can be used to extract 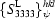 and the diffraction out-of-plane modulus 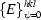, as in §4.2. By considering the macroscopic elastic anisotropy, the knowledge of 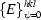 can be used to determine the mechanical Young’s modulus 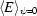 or the term 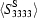.

(iii) By evaluating the intercepts on the 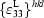 axis for 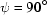, equation (13) can be used to determine the term 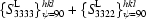, which, in this special case, can be used to quantify the in-plane biaxial modulus of the thin film 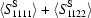.

In order to quantify the parameters 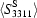, 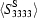 and 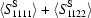, the macroscopic elastic anisotropy of the film must be considered. Furthermore, the determination of out-of-plane moduli 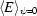 = 1/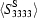 from the X-ray elastic constants 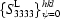 will be discussed.

### 4.4. Elastic modulus of 111 fibre-textured Cu thin film

**Figure 7:**
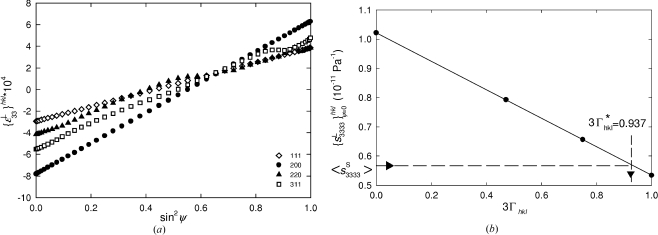
(*a*) Calculated X-ray elastic strains in a Cu thin film with a strong 111 fibre texture under equibiaxial stress of 100 MPa. (*b*) X-ray compliances 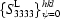 refined from (*a*), plotted as a function of 3Γ_*hkl*_. Since, for this special type of texture, the mechanical compliance 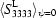 = 0.575 × 10^−11^ Pa^−1^, 3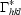 = 0.937 was extrapolated from the 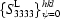 dependence on 3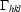.

In Fig. 7 ▶, calculated sin^2^ψ dependencies for a Cu thin film with a strong 111 fibre texture (Fig. 2 ▶) are presented. The plots were calculated supposing an in-plane isotropic stress of 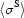 = 100 MPa and using the single-crystal elastic constants from Table 1 ▶.

The data in Fig. 7 ▶(*a*) were evaluated according the procedure described in §4.2 point (ii), and 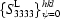 values were determined (Fig. 7 ▶ *b*). Using the ODF from Fig. 2 ▶, the out-of-plane mechanical compliance 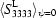 was also calculated [equations (3)–(7) [Disp-formula fd5] [Disp-formula fd6] [Disp-formula fd7]] with 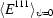 = 174 GPa. Comparison of the out-of-plane X-ray and mechanical compliances showed that 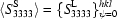 for 3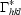 = 0.937. This result demonstrates that, in order to determine 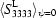 from 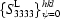 [*i.e.* to apply an opposite algorithm flow to that in Fig. 7 ▶(*b*)], it is necessary to know the value of 3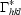, which is strongly texture dependent.

### 4.5. 3Γ^*^_hkl_–3Γ_*uvw*_ plot

**Figure 8:**
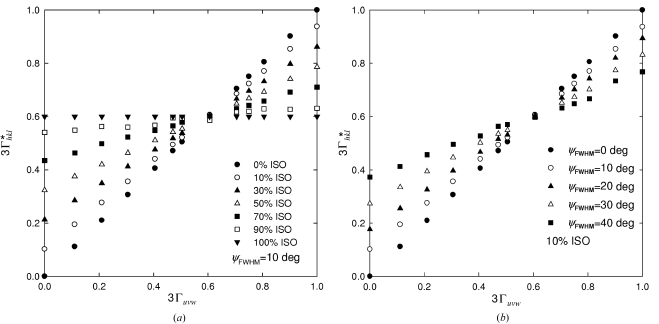
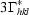
–3Γ_*uvw*_ plots indicating for which *hkl* reflection (and corresponding 3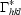 value) the X-ray elastic constants 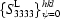 are equal to the mechanical constants 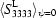. 3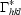 values are plotted as a function of the fibre-texture type expressed through Γ_*uvw*_. (*a*) The dependence of 3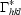 on the fraction of randomly oriented crystallites in the range 0–100%, plotted for various *uvw* textures with ψ_FWHM_ = 10°. (*b*) The dependence of 3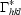 on ψ_FWHM_, plotted for various *uvw* textures supposing a 10% fraction of randomly oriented crystallites.

In the case of cubic *uvw* fibre-textured films with the fibre axis oriented perpendicularly to the substrate surface, the texture type will be further described using the parameters Γ_*uvw*_ defined as (Bollenrath *et al.*, 1967 ▶; Huang & Weaver, 2005 ▶) 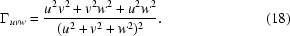
Supposing various *uvw* fibre textures (i) with a texture sharpness ψ_FWHM_ in the range 0–60° (Fig. 2 ▶), (ii) with 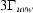 in the range 0–1 and (iii) with ISO in the range 0–100%, numerous ODFs were generated. Following the algorithm from §4.3 point (ii), 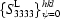 and 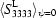 values were calculated numerically for materials with Zener’s anisotropy ratio in the range 0.36–9.95 (corresponding to KCl and Na). Then the mechanical and X-ray elastic constants were compared, with the aim of finding out for which 3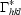 value 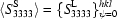. As a result 3
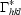
–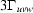 plots were constructed, indicating how 3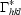 depends on the *uvw* fibre-texture type, on ψ_FWHM_ (Fig. 8 ▶ *b*) and on ISO (Fig. 8 ▶ *a*).

The 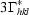
–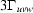 plots in Fig. 8 ▶ do not depend on the crystal elastic anisotropy of the thin-film material and represent therefore a certain type of universal plot valid for all materials. In the case of isotropic materials (like tungsten) where ZAR 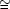 1, the choice of 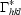 is arbitrary.

In Fig. 8 ▶(*a*), one can recognize that, for very strong *uvw* fibre textures with ψ _FWHM_ < 10° and a small or no fraction of randomly oriented crystallites, the X-ray elastic constants correspond approximately to the mechanical constants for 3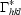 = 3Γ_*uvw*_. In other words, in order to determine the out-of-plane modulus of a thin film with a very strong *uvw* texture one has to characterize the X-ray elastic constants of the *uvw* reflections. For not very pronounced fibre textures, the 3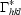 value must be selected from the intervals 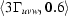 or 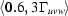 for thin films with Γ_*uvw*_ smaller or larger than 0.6, respectively. When the fraction of randomly oriented crystallites ISO increases, however, X-ray elastic constants of the *hkl* reflections for which 3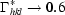 should be quantified (§4.2). Similarly, in Fig. 8 ▶(*b*), the decrease of the texture sharpness results in behaviour that is typical for elastic isotropic materials and 3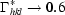.

In the case of sharp *uuu* or *u*00 fibre textures the search for an exact 3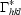 value is extremely important, because the application of the procedure from §4.2 (valid for elastic isotropic materials) could result in large errors when determining the out-of-plane moduli. For films with *uvw* fibre textures with 3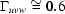, the procedure from §4.2 can still provide relevant results.

The results in Figs. 8 ▶(*a*) and 8 ▶(*b*) represent an example of the 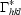
–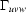 selection rule. In order to express the dependence of 3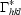 on 3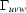, on ψ_FWHM_ and on ISO generally and in a ‘user-friendly’ way, the following empirical equation was derived: 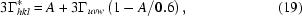
where *A* = (ψ_FWHM_ × 8.8 + ISO × 5.8 − ψ_FWHM_ × ISO × 0.083)/1000.

Equation (19) provides an easy way to determine 3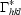 values considering fibre-texture parameters. The parameters ISO and ψ_FWHM_ in equation (19) can be obtained from pole figure data (Fig. 2 ▶), or they can be extracted from an ODF analysis of experimental pole figures. The ODF analysis is recommended especially in the case of strong mixed textures or texture gradients. It is important to note that in the quantification of the 3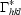 value using equation (19) the crystal elastic anisotropy does not play a role.

It is obvious that the considerations of §§4.1–[Sec sec4.5]4.5 can be extended to determine other mechanical elastic constants of thin films (*e.g.* in-plane biaxial moduli). Therefore, there is a need for a general approach when comparing 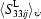 and 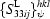 for various fibre-texture types and ψ angles.

The derived 3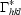 dependence on the texture parameters [equation (19)] based on the comparison of 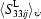 and 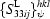 depends obviously on the supposed grain-interaction model. In the present case, the Hill grain-interaction model was used, and therefore the approach should be applied only to fibre-textured films that are assumed to obey that model. In the next section, the approach is demonstrated on the experimental characterization of fibre-textured Cu and CrN thin films.

## 5. Experimental procedure

### 5.1. Sample preparation

Cu and CrN thin films were deposited on Si(100) using the Balzers RCS coating system. In order to induce a measurable substrate curvature and to avoid a substrate plastic deformation, monocrystalline Si(100) wafers with thicknesses of 140 and 400 µm and lateral dimensions of 30 × 8 mm were chosen for the deposition of Cu and CrN films, respectively. The substrates were ultrasonically cleaned in acetone and alcohol, and Ar etched prior to the deposition. The Cu was deposited in an argon atmosphere at room temperature and then annealed at 673 K for 10 min in order to increase the residual stress (and substrate curvature) magnitude. The 3 µm-thick CrN thin film was deposited at a temperature of 623 K. The thicknesses of the Cu and CrN thin films (0.6 and 3 µm, respectively) were determined from the film cross sections using a scanning electron microscope. The thickness of the substrate was measured mechanically using a micrometre gauge with a precision of better than 1 µm.

### 5.2. Diffraction setup

The substrate curvature, elastic strain and texture of Cu and CrN on Si(100) were characterized in laboratory conditions using a Seifert 3000 PTS four-circle diffractometer. The setup comprised Cu *K*α radiation, polycapillary optics on the primary side, vertical Soller slits, a graphite monochromator and a scintillation detector on the secondary side. For the elastic strain and curvature characterization, beam sizes of 3.0 and 0.5 mm in diameter were chosen. The relatively large beam in the case of strain measurements enabled the assessment of volume-averaged properties. The elastic strains were determined with precision better than ±10%. The limited pole figure characterization was performed using the Schultz reflectivity technique with a beam of 2 mm in diameter, with the ψ range set to 0–80°. The rectangular samples were glued with just one of their narrower sides onto sample holders, in order to allow for free bending when the strain and the curvature were characterized in the diffractometer. For comparison, the texture of the films was also characterized using a Bruker GADDS system equipped with a two-dimensional detector, and the pole figures were identical to those obtained using the Seifert system.

### 5.3. Thin-film texture

**Figure 9:**
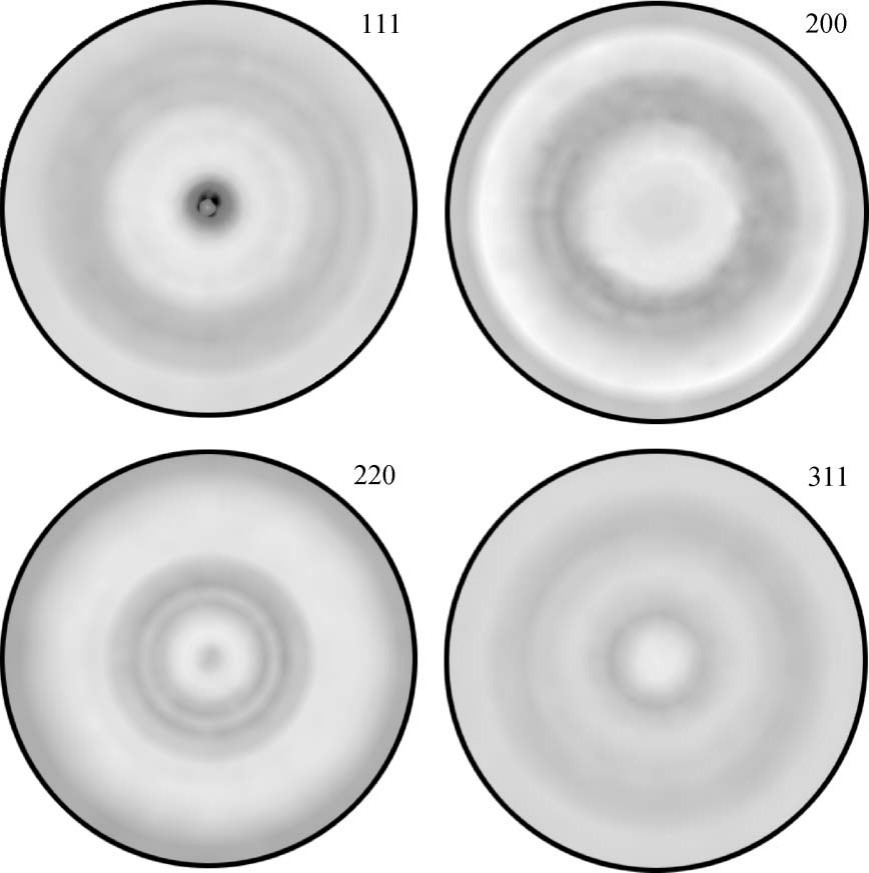
Experimental Cu 111, 200, 220 and 311 pole figures documenting the 111 fibre texture in the Cu thin film. The external ring corresponds to 80°.

**Figure 10:**
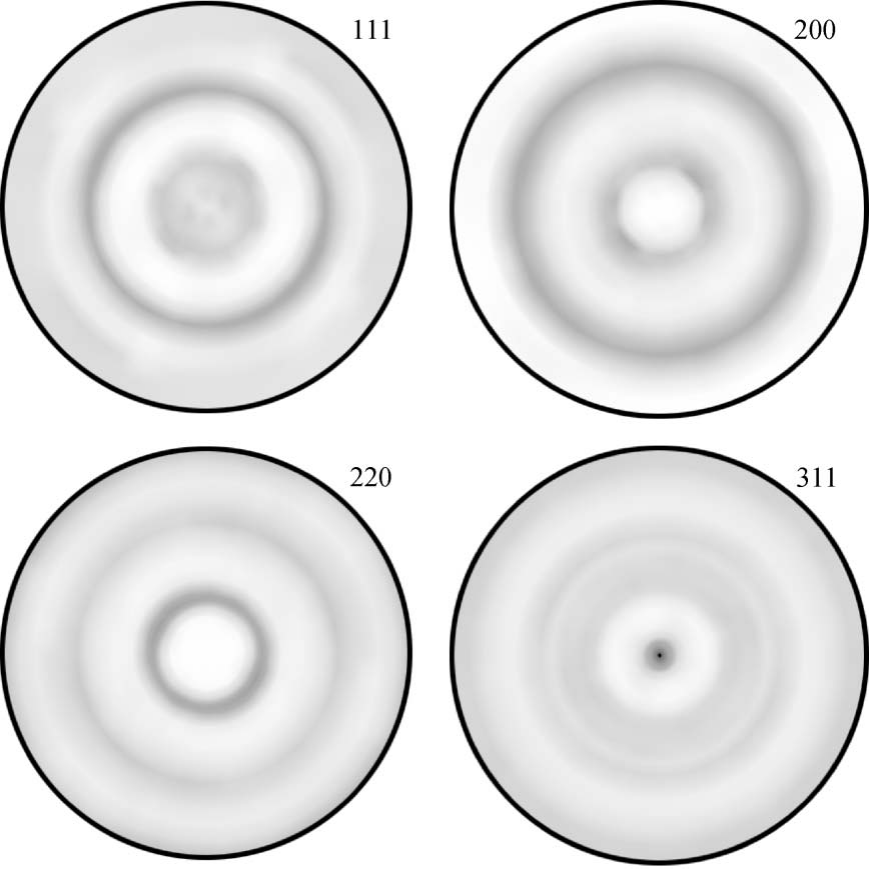
Experimental CrN 111, 200, 220 and 311 pole figures documenting a 311 fibre texture. The outer ring corresponds to 80°.

The texture in Cu and CrN thin films was characterized using pole figure measurements (Figs. 9 ▶ and 10 ▶). The orientation distribution function was then calculated from the experimental data in order to assess the proportion of randomly oriented crystallites ISO. The ODF analysis was performed using the commercial software *LaboTex* applying the ADC (arbitrarily defined cells) method (LaboSoft, 2006 ▶; Pawlik, 1986 ▶). In the case of Cu, one can easily identify a sharp 111 fibre texture (Fig. 9 ▶) with a width at half-maximum ψ_FWHM_ at the centre of the 111 pole figure of 14° and an ISO of 10%. For CrN, a 311 texture is visible in Fig. 10 ▶, with ψ_FWHM_ = 12° and an ISO of 13%.

The experimental parameters ψ_FWHM_ and ISO were used to determine 3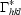 using equation (19). For the Cu and CrN thin films from Figs. 9 ▶ and 10 ▶, it was found that 3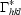 is equal to 0.89 and 0.51, respectively.

### 5.4. Macroscopic stress characterized by the X-ray diffraction substrate curvature technique

**Figure 11:**
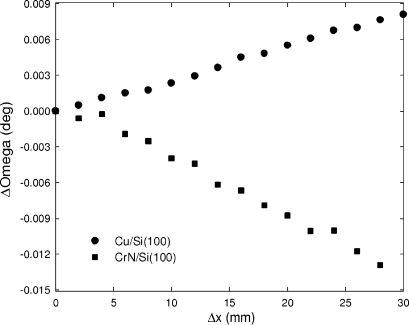
Plots of the Δω dependence on Δ*x* for the Cu/Si(100) and CrN/Si(100) samples. The results indicate different radii of curvature *R* of 2.193 and 3.572 m for Cu and CrN. The convex and the concave bending correspond to tensile and compressive stresses of 275.9 and −1415.9 MPa in Cu and CrN, respectively (Martinschitz *et al.*, 2006 ▶).

The pole figure measurements confirmed that the thin films are in-plane elastic isotropic. Since the films were unpassivated, the residual stress 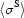 in the plane of the films was considered as equibiaxial and the out-of-plane components 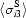 were neglected. The volume-averaged macroscopic stresses in Cu and CrN polycrystalline thin films were determined using the X-ray diffraction substrate curvature method (Stoney, 1909 ▶; Segmüller *et al.*, 1989 ▶; Zhao *et al.*, 2002 ▶; Keckes *et al.*, 2007 ▶). The quantification of the curvature was performed by the measurement of rocking curves of Si 400 reflections at different sample positions *x*_*i*_ as described in our previous work (Martinschitz *et al.*, 2006 ▶). In Fig. 11 ▶, the relative positions of the rocking curves (expressed through angle ω) on Δ*x* are presented for the Cu/Si(100) and CrN/Si(100) samples. The plots in Fig. 11 ▶ indicate a homogeneous curvature and residual stress across the sample. In practice, provided the sample homogeneity is not questionable, it would be enough to quantify the curvature from just a few measurement points.

The data in Fig. 11 ▶ were used to calculate the radius of curvature *R* according to
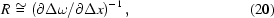
where 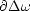/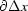 represents the slope of the linear dependencies (Martinschitz *et al.*, 2006 ▶). Applying *R*, it was possible to determine the macroscopic in-plane isotropic residual stress 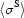 in the films using the Stoney (1909 ▶) formula 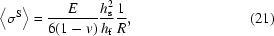
where *h*_s_ and *h*_f_ denote the substrate and film thicknesses, respectively, and the term *E*/(1 − ν) = 181 GPa is the biaxial modulus of the silicon substrate (Suresh & Freund, 2003 ▶). The macroscopic stress 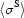 in the Cu and CrN films (Table 2 ▶) was determined with a precision of about ±5%.

### 5.5. Elastic strain in thin films

**Figure 12:**
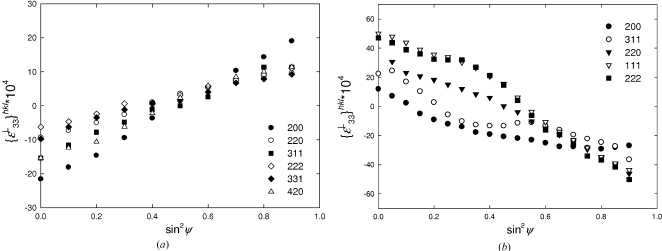
Measured X-ray elastic strains 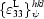 in Cu (*a*) and CrN (*b*) thin films as a function of the sample tilt angle ψ. Positive (*a*) and negative (*b*) slopes indicate tensile and compressive stresses in Cu and CrN, respectively. The strains were determined with a precision better than ±10%.

In Figs. 12 ▶(*a*) and 12 ▶(*b*) the X-ray elastic strains 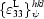 in the Cu and CrN films for different *hkl* reflections are presented as a function of the sample tilt angle ψ. The different crystal elastic anisotropy has the result that 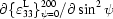 > 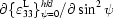 for Cu and 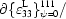 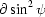 > 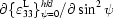 for CrN in Fig. 12 ▶. In the case of Cu, the dependencies are nearly linear, whereas for CrN films one can observe nonlinearities which can be attributed to the experimental errors and to gradients of strain, texture or unstressed lattice parameters. Especially in the case of hard coatings like CrN, nonlinearities (Fig. 12 ▶ *b*) are usual (*cf.* Donohue *et al.*, 1999 ▶; Göbel *et al.*, 2001 ▶). Although it has often been observed that polycrystalline samples do not exhibit perfectly linear experimental sin^2^ψ dependencies for *h*00 and *hhh* reflections, the application of the anisotropic Hill grain-interaction model to assess the mechanical behaviour of such samples has generally provided satisfactory results (van Houtte & De Buyser, 1993 ▶; Gnäupel-Herold *et al.*, 1998 ▶; Howard & Kisi, 1999 ▶). It is also known that results obtained using other more sophisticated models such as those of Kröner or Vook–Witt (Kröner, 1958 ▶; Leoni *et al.*, 2001 ▶; Welzel, 2002 ▶; Welzel *et al.*, 2005 ▶) are very close to those of Hill (van Houtte & De Buyser, 1993 ▶; Gnäupel-Herold *et al.*, 1998 ▶; Howard & Kisi, 1999 ▶, Welzel, 2002 ▶). Moreover, since the grain-interaction models represent idealized theories, it is very difficult to distinguish which model is applicable in the case of experimental data obtained from real materials. The precision of diffraction techniques is, moreover, often insufficient to distinguish between different models (Howard & Kisi, 1999 ▶). This is the case here also, since the precision of the X-ray elastic strain characterization was not better than 10%.

Since the films were polycrystalline, the methodology based on the anisotropic Hill grain-interaction model was used to extract mechanical elastic constants applying the formalism from §§2–4 [Sec sec4].

The plots in Fig. 12 ▶ illustrate that it was possible to perform lattice spacing measurements and to determine X-ray elastic strains at every sample tilt angle ψ, even for the Cu film with the strong 111 fibre texture. This fact indicates that there was a nonzero fraction of randomly oriented crystallites in the films. The lattice spacing measurements at arbitrary ψ angle were possible, however, only after the polycapillary optics and vertical Soller slits were installed and used (Welzel & Leoni, 2002 ▶). Therefore, the use of parallel X-ray optics seems to be an important prerequisite to apply successfully the method described in this work.

Another important prerequisite for the use of the new method is the fact that the strains should be analysed using a relatively large beam (3 mm in diameter in the present case). Only then can representative information on the average X-ray elastic strain be obtained.

### 5.6. Experimental Young’s moduli

**Figure 13:**
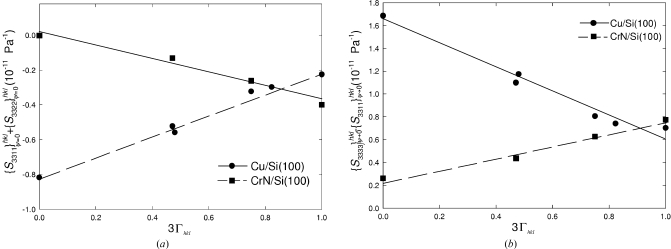
X-ray elastic constants 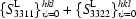 and 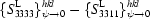 obtained by fitting equation (13) (§3.1) to the data from Fig. 12 ▶ and by evaluating the intercepts (*a*) and the slopes (*b*) under the consideration of the macroscopic stress 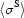 (§5.4).

The X-ray elastic constants 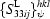 [equation (13) and §4.3] can be obtained by a numerical fitting of the experimental X-ray elastic strains 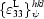 from Fig. 12 ▶, applying the macroscopic stress values 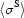 from Table 2 ▶. This type of analysis was performed in order to evaluate (i) 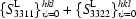 from the intercepts on the 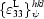 axis and (ii) 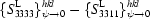 from the slopes in Fig. 12 ▶. In Figs. 13 ▶(*a*) and 13 ▶(*b*), the fitted parameters 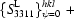 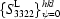 and 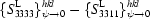 from Fig. 12 ▶ are presented as a function of 3Γ_*hkl*_ for the Cu and CrN thin films. These parameters differ for various *hkl* reflections, which is the consequence of the crystal elastic anisotropy.

The 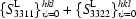 and 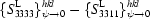 dependencies on 3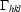 from Fig. 13 ▶ were approximated by linear dependencies and the results are presented in Table 2 ▶. By easy calculus it was possible to derive also the dependence of 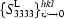 on 3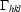 (Table 2 ▶). Considering the macroscopic elastic anisotropy and by applying the 3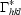 values from §5.3 one can determine an inverse out-of-plane X-ray elastic modulus 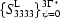 which is equal to the mechanical compliance 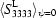. The out-of plane Young’s modulus can then be easily determined as follows: 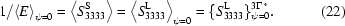
The experimental out-of-plane Young’s moduli of Cu and CrN thin films were found to be 169.40 and 244.87 GPa. The results are comparable to the experimental data obtained using other techniques (Hong *et al.*, 2005 ▶; Sue *et al.*, 1994 ▶; Lee *et al.*, 2008 ▶).

### 5.7. Error discussion

The accuracy with which the out-of-plane Young’s moduli were determined using the new algorithm is influenced by numerous factors. The approach is based on the combined application of well established techniques, sin^2^ψ and X-ray diffraction substrate curvature, the experimental accuracies of which have been discussed in numerous papers (*e.g.* Noyan & Cohen, 1987 ▶; Winholz & Cohen, 1988 ▶; Zhao *et al.*, 2002 ▶). The combination of the two techniques can in the worst case result in the accumulation of experimental errors.

The accuracy of the sin^2^ψ technique was assessed by Noyan & Cohen (1987 ▶) and by Winholz & Cohen (1988 ▶). Depending on numerous parameters, such as sample quality, diffraction system, number of measured reflections, scattering intensity and measurement time (Noyan & Cohen, 1987 ▶; Winholz & Cohen, 1988 ▶), the precision is usually below ±15%. Moreover, the exactness of the elastic strain characterization can be improved by increasing the number of measured reflections (Fig. 13 ▶). In the present case, since the measurements were performed using a commercial diffractometer, the precision was about ±10%

The exactness of the macroscopic stress characterization is extremely important since the stress is used to divide the experimental strain values. For this reason, not only the substrate curvature but also the film and the substrate thickness must be determined with a high precision. In the present case, the macroscopic stresses were determined with a precision of ±5%. Experimental elastic strain and macroscopic stress data are combined in Fig. 13 ▶, whereby the linear dependencies provided coefficients of determination *R* ^2^ larger than 0.9.

The data in Fig. 13 ▶ were used to extract 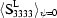 parameters and finally also elastic moduli. It can be therefore supposed that the moduli in Table 2 ▶ were determined in the present case with a precision better than 15%.

Fig. 13 ▶ implies good control over the reliability of the technique proposed here. If the dependence of 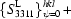 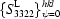 and 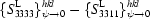 on 3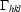 is not linear and the *R* ^2^ factors are smaller than 0.8, the technique will not provide reliable data.

Moreover, the simultaneous application of sin^2^ψ and X-ray diffraction curvature techniques should be performed on a representative sample region without strong gradients in microstructure and in stresses. By extending the curvature characterization to a large Δ*x* range (Martinschitz *et al.*, 2006 ▶), it is possible to analyse if the curvature and the stress are homogeneous. The strain measurements should be performed in the region for which the curvature as well as the film and the substrate thickness are known. When performing the strain characterization, however, a large beam ensuring good statistics is required.

Another source of error could reside in the parameter 3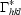. The parameter can be quantified exactly using a numerical ODF analysis of the texture data or estimated from the pole figure plots. The higher the crystal elastic anisotropy of the materials, the more significantly the 3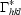 inaccuracy will contribute to the errors when determining the moduli.

Similarly, the present approach supposes that thin films possess a certain type of fibre texture, which occurs, for instance, in annealed metallic films (where the film thickness is comparable to the crystallite thickness; Eiper *et al.*, 2007 ▶). Since some thin films possess very complex fibre textures (with strong gradients), a careful ODF analysis must be performed before comparing 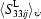 and 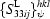.

Incorrect values of moduli will be obtained when the monocrystalline substrate under the film is plastically deformed. In that case the Stoney formula does not hold. For this reason, it is important to pay significant attention to the sample preparation.

An important assumption made in this work is that the mechanical behaviour of the polycrystalline films can be described using the Hill grain-interaction model. This is generally not the case (for instance, in epitaxial thin films where behaviour according the Voigt model can be expected). Therefore, the proposed method cannot be applied automatically. It is always important to analyse the microstructure of the film. In the case of film materials with unknown grain-interaction mechanism, it is recommended to perform a comparative characterization using other techniques, such as nanoindentation. Non­linearities in the 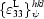
–sin^2^ψ dependencies are often an important indication that a polycrystalline film does not obey the Hill grain-interaction model. As already mentioned, nonlinearities can be attributed also to gradients of strain, texture or unstressed lattice parameters, or to plasticity in the film. In the case of strong nonlinearities, the method should not be applied. Although in the present case (Fig. 12 ▶ *b*) the 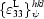
–sin^2^ψ dependencies are not perfectly linear, the results obtained from the CrN film are comparable to results obtained using other techniques (Sue *et al.*, 1994 ▶; Lee *et al.*, 2008 ▶).

## 6. Conclusions

A new method to determine elastic moduli of thin films in a contactless manner using X-ray light was proposed. Supposing the Hill grain-interaction model, it was demonstrated that X-ray elastic constants can be used to determine mechanical elastic constants of cubic thin films with strong fibre textures. For this purpose, numerically calculated X-ray elastic constants of polycrystalline films were compared with their mechanical counterparts. The results demonstrate that the algorithm to determine the mechanical elastic constants strongly depends on the fibre-texture type, the texture sharpness, the number of randomly oriented crystallites in the polycrystalline aggregate and the assumed grain-interaction model. For this purpose, a universal plot (and equation) was derived. The method was used to quantify out-of-plane Young’s moduli of Cu and CrN fibre-textured thin films with satisfactory results.

## Figures and Tables

**Table 1:** Single-crystal elastic constants (in 10^−3^ GPa^−1^) of Cu and CrN at room temperature and the Zener (1948 ▶) anisotropy ratio (ZAR) defined by equation (8) (Suresh & Freund, 2003 ▶; Birkholz, 2006 ▶)

Material	*S*_1111_	*S*_1122_	*S*_1212_	ZAR
Cu	15.00	−6.28	3.32	3.21
CrN	1.860	−0.09	2.84	0.34

**Table 2:** An experimental algorithm to determine out-of-plane mechanical moduli of fibre-textured thin films is presented The macroscopic stress 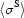 was determined using the curvature measurement (§5.4 and Fig. 11 ▶). The elastic strain 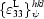 dependencies on sin^2^ψ (§5.5 and Fig. 12 ▶) were analysed in order to evaluate the intercepts 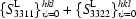 and slopes 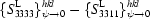 for *ψ* 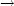 0. The factor 3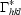 indicates for which value of the X-ray anisotropic factor the X-ray and mechanical elastic constants are equal (§5.3). The compliances and the moduli were then determined using equation (22).

	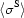	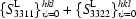	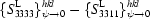	3 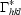	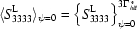	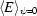
	(MPa)	(10^−11^ Pa^−1^)	(10^−11^ Pa^−1^)		(10^−11^ Pa^−1^)	(GPa)
Cu	275.9	0.62 × 3Γ_*hkl*_ − 0.82	−1.04 × 3Γ_*hkl*_ + 1.65	0.89	0.5903	169.40
CrN	−1415.9	−0.43 × 3Γ_*hkl*_ + 0.046	0.51 × 3Γ_*hkl*_ + 0.24	0.51	0.4084	244.87
